# Transcriptomic responses of water buffalo liver to infection with the digenetic fluke *Fasciola gigantica*

**DOI:** 10.1186/s13071-017-1990-2

**Published:** 2017-02-01

**Authors:** Fu-Kai Zhang, Xiao-Xuan Zhang, Hany M. Elsheikha, Jun-Jun He, Zhao-An Sheng, Wen-Bin Zheng, Jian-Gang Ma, Wei-Yi Huang, Ai-Jiang Guo, Xing-Quan Zhu

**Affiliations:** 1 State Key Laboratory of Veterinary Etiological Biology, Key Laboratory of Veterinary Parasitology of Gansu Province, Lanzhou Veterinary Research Institute, Chinese Academy of Agricultural Sciences, Lanzhou, Gansu Province 730046 People’s Republic of China; 2 Faculty of Medicine and Health Sciences, School of Veterinary Medicine and Science, University of Nottingham, Sutton Bonington Campus, Loughborough, LE12 5RD UK; 3 College of Animal Science and Technology, Guangxi University, Nanning, Guangxi Zhuang Autonomous Region 530005 People’s Republic of China; 4 Jiangsu Co-innovation Center for Prevention and Control of Important Animal Infectious Diseases and Zoonoses, Yangzhou, Jiangsu Province 225009 People’s Republic of China

**Keywords:** *Fasciola gigantica*, Immunomodulation, RNA-sequencing, Transcriptome, Water buffalo

## Abstract

**Background:**

*Fasciola gigantica*, the tropical liver fluke, infects buffaloes in Asian and African countries and causes significant economic losses and poses public health threat in these countries. However, little is known of the transcriptional response of buffaloes to infection with *F. gigantica*. The objective of the present study was to perform the first transcriptomic analysis of buffalo liver infected by *F. gigantica*. Understanding the mechanisms that underpin *F. gigantica* infection in buffaloes will contribute to our ability to control this parasite.

**Methods:**

We challenged buffaloes with 500 viable *F. gigantica* metacercariae and collected liver samples through a time course at 3, 42 and 70 days post-infection (dpi). Then, we performed gene expression analysis on liver samples using RNA sequencing (RNA-Seq) Illumina technology and confirmed the RNA-Seq data by quantitative RT-PCR analysis.

**Results:**

Totals of 496, 880 and 441 differentially expressed transcripts were identified in the infected livers at 3, 42 and 70 dpi, respectively. Gene Ontology (GO) and Kyoto Encyclopedia of Genes and Genomes (KEGG) analysis revealed that transcriptional changes in the liver of infected buffaloes evolve over the course of infection. The predominant response of buffaloes to infection was mediated by certain pathways, such as MHC antigen processing and presentation, Toll-like receptor 4 (TLR4), transforming growth factor beta (TGF-β), and the cytochrome P450. Hepatic drug metabolizing enzymes and bile secretion were also affected.

**Conclusions:**

*Fasciola gigantica* can induce statistically significant and biologically plausible differences in the hepatic gene expression of infected buffaloes. These findings provide new insights into the response of buffaloes to *F. gigantica* over the course of infection, which may be useful in determining pathways that can modulate host-parasite interaction and thus potentially important for clearance of the parasite.

**Electronic supplementary material:**

The online version of this article (doi:10.1186/s13071-017-1990-2) contains supplementary material, which is available to authorized users.

## Background

Fasciolosis is a serious liver disease caused by infection with the digenetic trematodes *Fasciola hepatica* and *F. gigantica* in temperate and tropical countries, respectively [1]. These flukes enter the definitive host, such as cattle, buffaloes, sheep and goats, orally and migrate towards the liver *via* the peritoneal cavity. Clinically affected animals exhibit a reduction in the growth rates, development and productivity, and in severe cases fasciolosis may lead to death [2]. Fasciolosis causes significant economic losses in livestock industry [3] and is a serious public health problem [4] by causing liver fibrosis, cirrhosis and cancer in humans [5]. Adult flukes have been recovered from the bile duct of humans from almost all continents [6, 7]. Reported estimates indicate that up to 17 million people are infected worldwide and that about 91 million are at risk [8].

*Fasciola gigantica* (tropical liver fluke), the major fluke infecting ruminants in Asia and Africa, can adversely affect the weight gain, feed conversion efficiency and reproduction of the affected buffaloes, imposing a serious threat to buffalo farming in these countries [9]. Current methods to control liver fluke infection rely on the use of fasciocidal drugs. However, the escalating anthelmintic resistance (AR) in ruminants has become a major concern [10, 11]. In an effort to discover novel alternative fasciocidal drugs, the anthelmintic efficacy of some medicinal plants against liver flukes have been tested [12, 13]. Also, many immunization trials in mice [14], rabbits [15], sheep [16, 17], goats [18] and cattle [19] exploiting various antigens and adjuvant systems [20] have been reported, but unfortunately these trials did not evoke adequate immune response to protect against challenge infection [18].

The liver fluke *F. hepatica* infection induces a dominant Th2/T-regulatory type immune response [21] and is known to modulate the host immune responses by various mechanisms, including the production of immune-suppressive cytokines and the alternative activation of macrophages [22], the increase of regulatory T cells [23] and the modulation of differentiation and function of dendritic cells [24, 25]. In contrast, the immunity elicited against *F. gigantica* infection is a mix of Th1/Th2 response with a predominance of a Th2-biased pattern [26, 27]. Recent studies have employed RNA sequencing (RNA-seq) to elucidate the expressions of genes associated with host’s immune responses, metabolism and transcriptomic changes in peripheral blood mononuclear cells (PBMCs) [28–30]. However, much still is unknown regarding the host immune response mechanisms against *F. gigantica* and the extent to which this response contributes to the resilience of buffaloes compared to certain cattle breeds [31, 32].

Herein, we utilized RNA-Seq to determine the transcriptional profiles of the liver of buffaloes infected with *F. gigantica*. We compared the differential gene expression of liver from infected buffaloes to that from uninfected controls. As the first report of a transcriptome analysis of buffalo liver during *F. gigantica* infection, the data presented here provide new insights into the response of buffalo to *F. gigantica* and revealed distinct pathways that are dysregulated by *F. gigantica* in the liver of infected buffaloes.

## Methods

### Metacercariae

Eggs of *F. gigantica* were collected from the gall-bladder of naturally infected buffaloes from Guangxi Zhuang Autonomous Region, PR China, and incubated at 29 °C for 11 days. The newly-hatched miracidia were used to infect *Galba pervia* snails (3–5 miracidia/snail) maintained in tissue culture plastic plate for 2 h and then infected snails were incubated in order to allow the miracidium stage to develop to sporocyst, redia, daughter redia and finally to cercariae. After 42 days, fully-developed cercariae emerged from the snails and were harvested and developed into metacercariae on 5 × 5 cm cellophane sheets. The metacercariae on cellophane sheets were washed several times with phosphate buffered saline (PBS) and were used immediately to infect buffaloes as described previously [33].

### Animals and experimental infection

Eighteen buffaloes, 8–10-month-old, were purchased from a water buffalo farm in Guangxi Zhuang Autonomous Region, PR China. Animals were randomly divided into two groups: (i) nine for the non-infected control group and (ii) nine for the infected group. Each group was further subdivided into 3 subgroups, each of 3 buffaloes. To rule out any prior infection with *F. gigantica*, faecal examination and ELISA testing using IgG and IgM antibodies against *F. gigantica* were performed [34]. Also, after an acclimatization period of 2 weeks, all buffaloes were treated with triclabendazole 5% w/v oral suspension in order to eliminate any liver flukes. After four weeks of the withdrawal time, nine buffaloes were infected orally with 500 viable metacercariae per animal, whereas control animals were mock-inoculated with 0.85% NaCl solution without metacercariae [31]. At 3, 42 and 70 days post-infection (dpi), three animals from each of the infected and control groups were sacrificed and their livers were collected and stored at -80 °C until analysis. Liver was selected because it is the target organ and preferable habitat of *F. gigantica* flukes in the definitive host (buffaloes) and in the mean time it performs many vital physiological, metabolic and immunological functions in the body [35]. At necropsy, whole blood samples of all animals were collected aseptically into tubes without anticoagulant and were separated by centrifugation for collection and testing of the sera. *Fasciola gigantica* infection was also confirmed by observing gross pathological lesions associated with the presence of adult flukes in the liver of infected animals.

### RNA preparation

Total RNA was extracted from individual liver samples using Trizol reagent according to the manufacturer’s instructions (Invitrogen Co. Ltd, San Diego, USA). All RNA samples were treated with 20 units of RQ1 RNase-Free DNase (Promega, Madison, USA) to remove any residual genomic DNA according to the manufacturer’s protocol. Agilent Bioanalyzer 2100 (Agilent Technologies, CA, USA) and NanoPhotometer® spectrophotometer (IMPLEN, Westlake Village, CA, USA) were used to evaluate the integrity and purity of RNA samples, respectively.

### Library preparation, clustering and transcriptome sequencing

RNA (3 μg) of each liver sample was used for the preparation of RNA libraries. Eighteen sequencing libraries were constructed using NEBNext® Ultra™ RNA LibraryPrep Kit for Illumina® (NEB, Ipswich, MA, USA) following the manufacturer’s protocol and index codes were added to attribute the sequences to the corresponding sample. Briefly, mRNA was purified from total RNA using poly-T oligo-attached magnetic beads. Fragmentation was carried out using divalent cations under elevated temperature in NEBNext First Strand Synthesis Reaction Buffer (5×). First strand cDNA was synthesized using random hexamer primer and M-MuLV Reverse Transcriptase (RNase H-). Second strand cDNA synthesis was subsequently performed using DNA Polymerase I and RNase H. In order to select cDNA fragments ranging from 150 bp to 200 bp, the library fragments were purified with AMPure XP system (Beckman Coulter, Beverly, USA). Three μl USER Enzyme (NEB, Ipswich, MA, USA) were used with size-selected, adaptor-ligated cDNA at 37 °C for 15 min followed by 5 min at 95 °C before PCR. Then, the PCR was carried out with Phusion High-Fidelity DNA polymerase, universal PCR primers and index (X) Primer. PCR products were purified (AMPure XP system) and library quality was evaluated on the Agilent Bioanalyzer 2100 system. The clustering of the index-coded samples was performed on a cBot Cluster Generation System using TruSeq PE Cluster Kit v3-cBot-HS (Illumia) according to the manufacturer’s instructions.

### Data analysis

Raw reads of fastq format were processed through in-house Perl scripts. Clean reads were obtained by removing reads adapters, ploy-N containing reads and low quality reads from raw data. At the same time, Q20, Q30 and GC content of clean data were calculated. All downstream analyses were based on the clean data with high quality. *Bubalus bubalis* genome was used as the reference genome and gene model annotation files were downloaded from the water buffalo genome website (http://www.ncbi.nlm.nih.gov/genome/?term=Bubalus+bubalis). Index of the reference genome was built using Bowtie v2.2.3 and paired-end clean reads were aligned to *Bubalus bubalis* reference genome using TopHat v2.0.12. We selected TopHat as the mapping tool because it can generate a database of splice junctions based on the gene model annotation file and provide a better mapping result than other non-splice mapping tools [36]. HTSeq v0.6.1 was used to count the read’s numbers mapped to each gene. Fragments Per Kilobase of transcript sequence per Millions base pairs sequenced (FPKM) of each gene was calculated for estimating gene expression levels. Differential expression analysis of two groups (three replicates per group) was performed using the DESeq R package (1.18.0) [37]. The resulting *P*-values were adjusted using the Benjamini and Hochberg’s approach for controlling the false discovery rate. Gene expression differences were considered significant if the adjusted *P*-value was < 0.05 and > 1.5-fold change was observed in expression levels.

### Gene ontology (GO) and Kyoto encyclopedia of genes and genomes (KEGG) analysis

Gene Ontology (GO) enrichment analysis of differentially expressed genes (DEGs) was implemented using the GOseq R package [38]. GO enrichment analysis was performed by collating all the GO terms that were significantly enriched in the identified DEG, and followed by filtering the DEGs based on the biological functions. All DEGs were mapped to GO terms in the database (http://www.geneontology.org/), and then gene numbers were calculated for every term using the hypergeometric test in order to obtain significantly enriched GO terms for DEGs; these were compared to the genomic background. GO terms with corrected *P-*value less than 0.05 were considered significantly enriched by DEGs. KOBAS software was used to perform pathway enrichment analysis and to test the statistical enrichment of the DEGs in KEGG (http://www.genome.jp/kegg/) [39, 40]. This analysis was used to identify significant enrichment of genes involved in metabolic or signalling pathways. AnimalTFDB (http://www.bioguo.org/AnimalTFDB/) was employed to identify and classify the transcriptional factors (TFs) in the genome of water buffalo.

### qRT-PCR verification of RNA-Seq expression patterns

Total RNA was isolated from infected livers and non-infection controls at 3, 42 and 70 dpi using RNeasy Mini Kit (QIAGEN Gmbh, Hilden, Germany). DNase-digested total RNA (1 μg) was reverse-transcripted to single strand cDNA using the RT^2^ First Strand Kit (QIAGEN Science, Maryland, USA) according to the manufacturer's protocol. RT^2^ SYBR® Green ROX qPCR Mastermix (QIAGEN Gmbh, Hilden, Germany) was used to perform qRT-PCR reaction on ABI’s real-time PCR cycler, the ABI 7500, according to the manufacturer’s instructions. Eleven genes were randomly selected for qRT-PCR verification. Forward (F) and reverse (R) primer pairs used to amplify genes of interest in the qRT-PCR reactions are listed in Table 1. The amplification reactions were performed using following conditions: 95 °C for 10 min, 40 cycles of 95 °C for 15 s, 60 °C for 1 min. Melting curve analysis was carried out using following conditions: 1 min at 95 °C, 65 °C for 2 min and progressive increase from 65 °C to 95 °C to ensure that a single product was amplified in each reaction.

**Table 1:** Primers used in the quantitative RT-PCR in the present study

Primer name	Primer sequence (5' to 3')	Length of qPCR products (bp)
CYTP450F	AGCAGCAGACAACATCAACCA	122
CYTP450R	CAATCGTCCTCTTCCCCATC	
IL7RF	CAGAGGAGAGTGAGAAGCAGAGG	275
IL7RR	GGGTTGGAATGGAAATGGAG	
NKRF	GCAATGTCAGCAATCAAGTCAG	174
NKRR	TCCTCTTCTTCCTCCACACACA	
IL1R2F	TGTGAGGGGAACTCGCTTACTC	105
IL1R2R	GTGATGTTGTATTGCCTGCCTTT	
BUT-LF	AAGAGAGAGCTTGCCAGAAGGA	143
BUT-LR	GATAAGACGAGGTTGGGGTGAG	
IP6K3F	CACGGCAGCAGTGTCTTCA	94
IP6K3R	CATCGTAGGTGGTGTGTTCATTC	
CD1EF	TTCCAGCCAAATCACAGACAA	133
CD1ER	TCACTTCCCCTCCACTTCTCC	
CTSHF	GCTTCAGTCACCCAACTCCAC	118
CTSHR	ATACCAGCCAGCATCCCTACA	
SOD3F	GACTGCCTCCTCTCTGCCTTT	150
SOD3R	TGTCCCCAGCAACTCTTTTC	
NCF4F	CTGTTTCCTCGCCTTGTTCC	273
NCF4R	CCTCCCTTCACCGCTTACTTAC	
βF	GGACTTCGAGCAGGAGATGG	138
βR	AGGAAGGAAGGCTGGAAGAGA	
b561F	GTATGTACCGAGGCGGCATT	148
b561R	ACTTTGGTGGTGCGTTTGG	

## Results

### Confirmation of *F. gigantica* infection in buffaloes

*Fasciola gigantica* infection was confirmed in all challenged buffaloes by observing gross pathological lesions and adults *F. gigantica* flukes (Additional files 1 and 2: Figure S1 and Figure S2). Livers of the control uninfected buffaloes appeared normal and were free of any *F. gigantica* flukes. Serological testing using ELISA confirmed the infection in all animals challenged with metacercariae at 42 and 70 dpi.

### Transcriptomic features of buffalo livers following *F. gigantica* infection

**Fig. 1:**
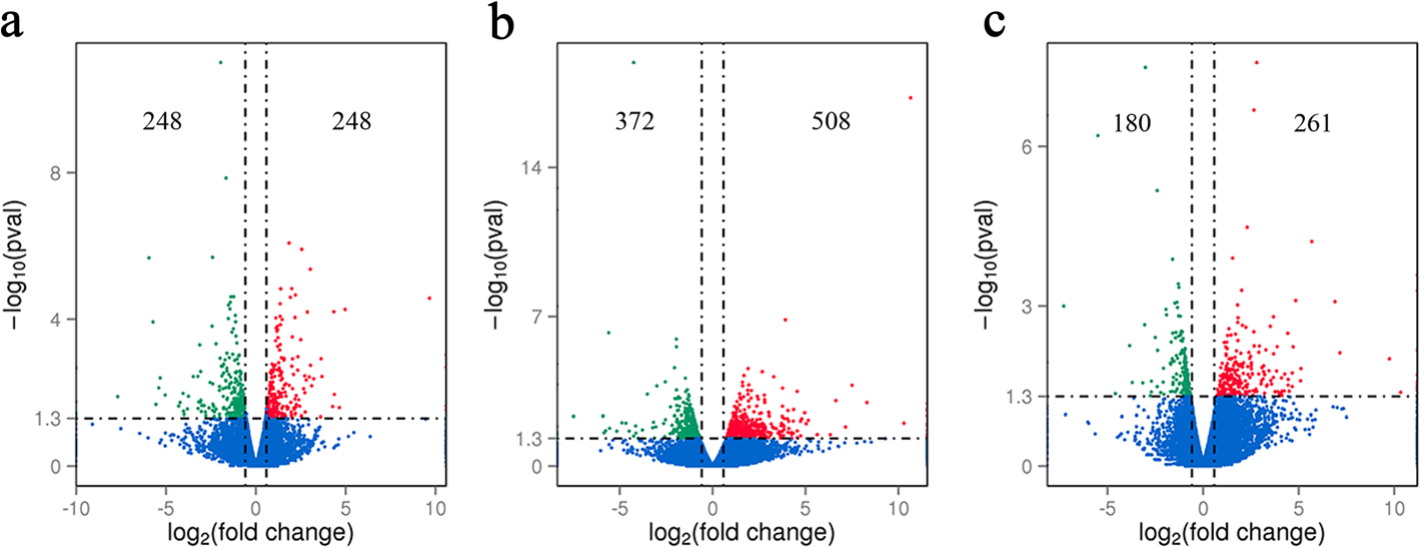
Volcano map of the differentially expressed genes between infected and control buffaloes. Significantly differentially expressed genes are shown as *red* (up) or *green* (down) dots. No significant difference between the expressions of genes is indicated by *blue* dots. Ordinate represents the magnitude of gene expression changes. The x-axis represents the value of log_2_(fold change) and the y-axis shows the value of -log_10_(pval). **a**, **b** and **c** represent differentially expressed genes at 3, 42 and 70 days post-infection, respectively

**Fig. 2:**
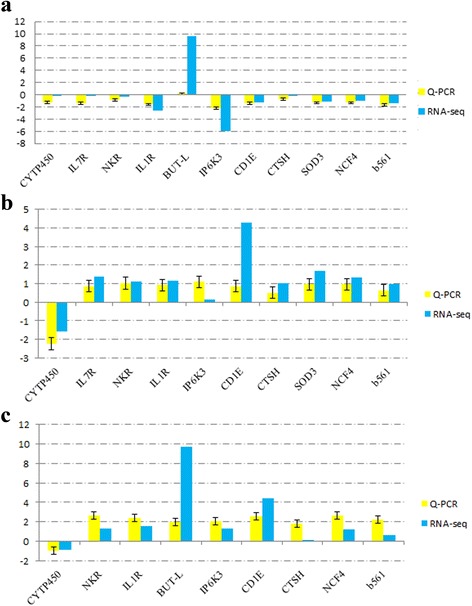
Verification of the gene expression by qRT-PCR. Eleven genes were selected randomly for validation of the RNA-seq data. Data of RNA-seq verified by qRT-PCR at 3 (**a**), 42 (**b**) and 70 (**c**) days post-infection

**Fig. 3:**
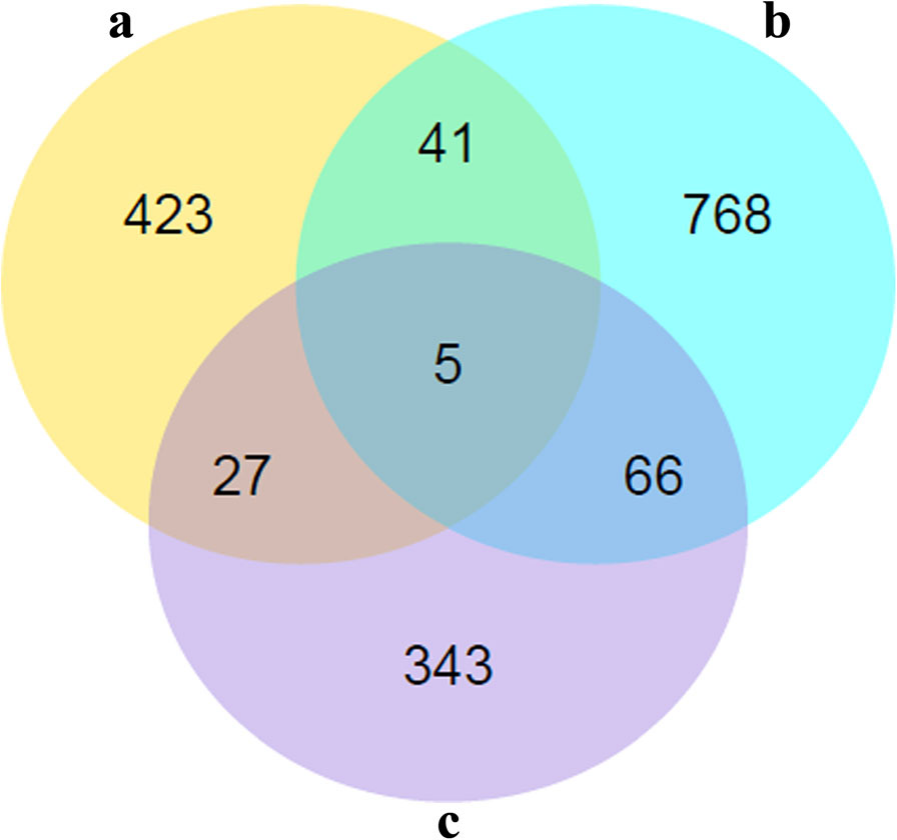
Venn diagram showing the overlap of the differentially expressed genes between *Fasciola gigantica*-infected liver sample groups at 3 (**a**), 42 (**b**) and 70 (**c**) days post-infection. Transcripts that are common to multiple time points are shown by the overlap

Over 58,000,000 raw reads (Additional file 3: Table S1) were obtained from each liver sample. The RNA-seq raw data are available at NCBI (accession no: PRJNA341921). More than 53,000,000 clean reads were obtained after removing low quality reads and adaptors. More than 70% clean reads were distributed in exon regions and the rest in introns or intergenics. A total of 496, 880 and 441 transcripts were identified as differentially expressed at 3, 42 and 70 dpi, respectively, compared to non-infected control groups (Fig. 1). The RNA-seq results were confirmed by qRT-PCR (Fig. 2). This analysis revealed five DEGs in all animal groups including butyrophilin subfamily 1 member A1-like (Gene ID: 102404197), myeloid-associated differentiation marker-like (Gene ID: 102406172), phosphoserine aminotransferase 1 (Gene ID: 102410803), and two new genes (Fig. 3).

### GO classification

**Fig. 4:**
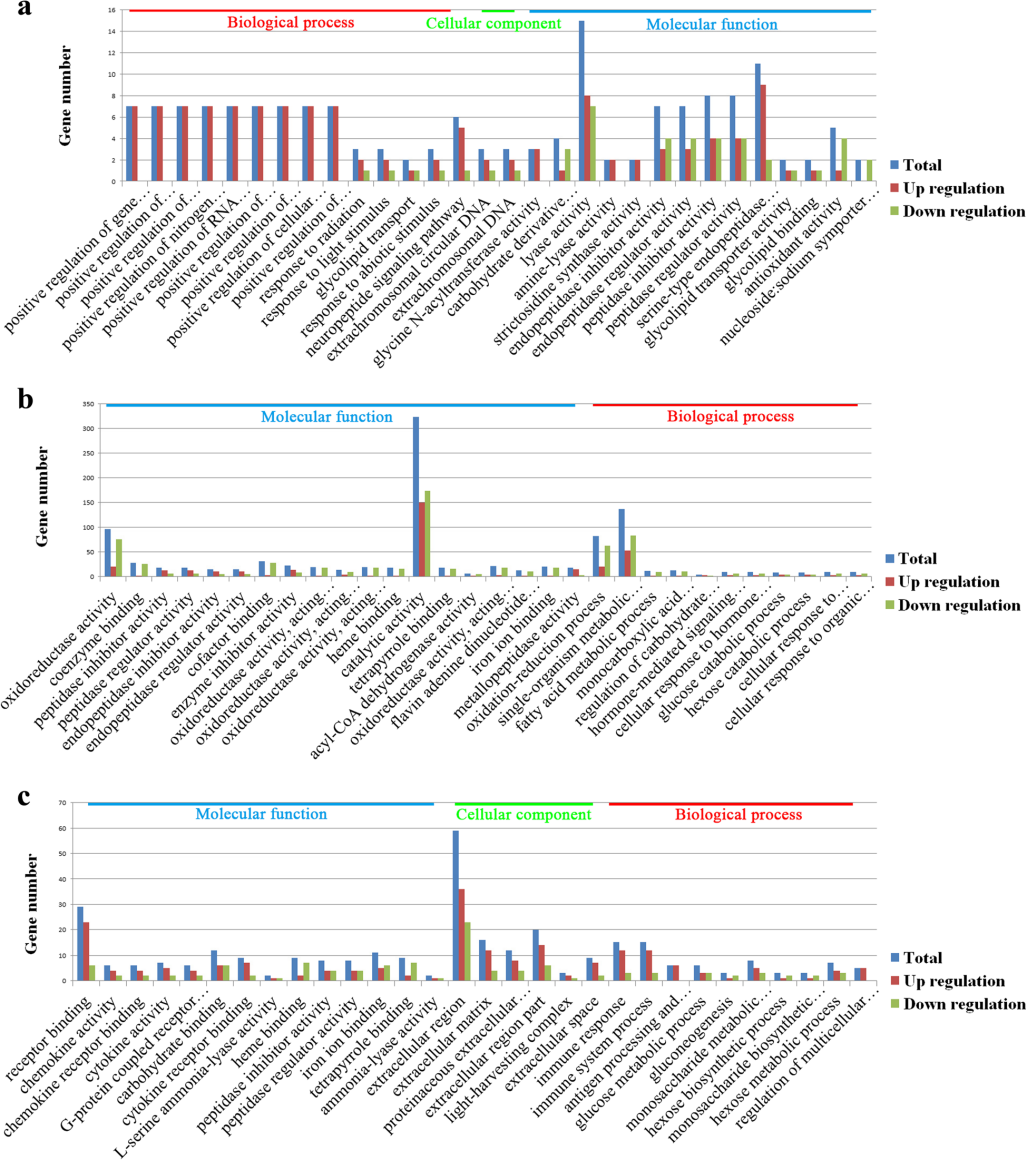
Differentially expressed GO terms. Differentially expressed genes (DEGs) were classified into three main categories: molecular function, cellular component and biological process. The identified functions and the corresponding numbers of DEGs for each GO category are shown. **a** Top 30 DE molecular function, cellular component and biological process in A2T (infected) *vs* A2C (control) at 3 dpi. **b** Top 30 DE molecular function and biological process in A5T (infected) *vs* A5C (control) at 42 dpi. **c** Top 30 DE molecular function, cellular component and biological process in A6T (infected) *vs* A6C (control) at 70 dpi

GO enrichment analysis (www.geneontology.org) revealed top 30 significant differentially expressed GO terms that were classified into “molecular function”, “biological process”, and “cellular component” as shown in Fig. 4. At 3 dpi, several GO terms classified in biological process showed upregulation. Only two significant GO terms were classified under “cellular component”, including “extra chromosomal circular DNA” and “extra chromosomal DNA”. While at 42 dpi, top 30 significant differentially expressed GO terms were only classified into “molecular function” and “biological process”. The “immune response”, “immune system process” and “cytokine activity” were significantly enriched at 70 dpi.

### KEGG analysis

**Fig. 5:**
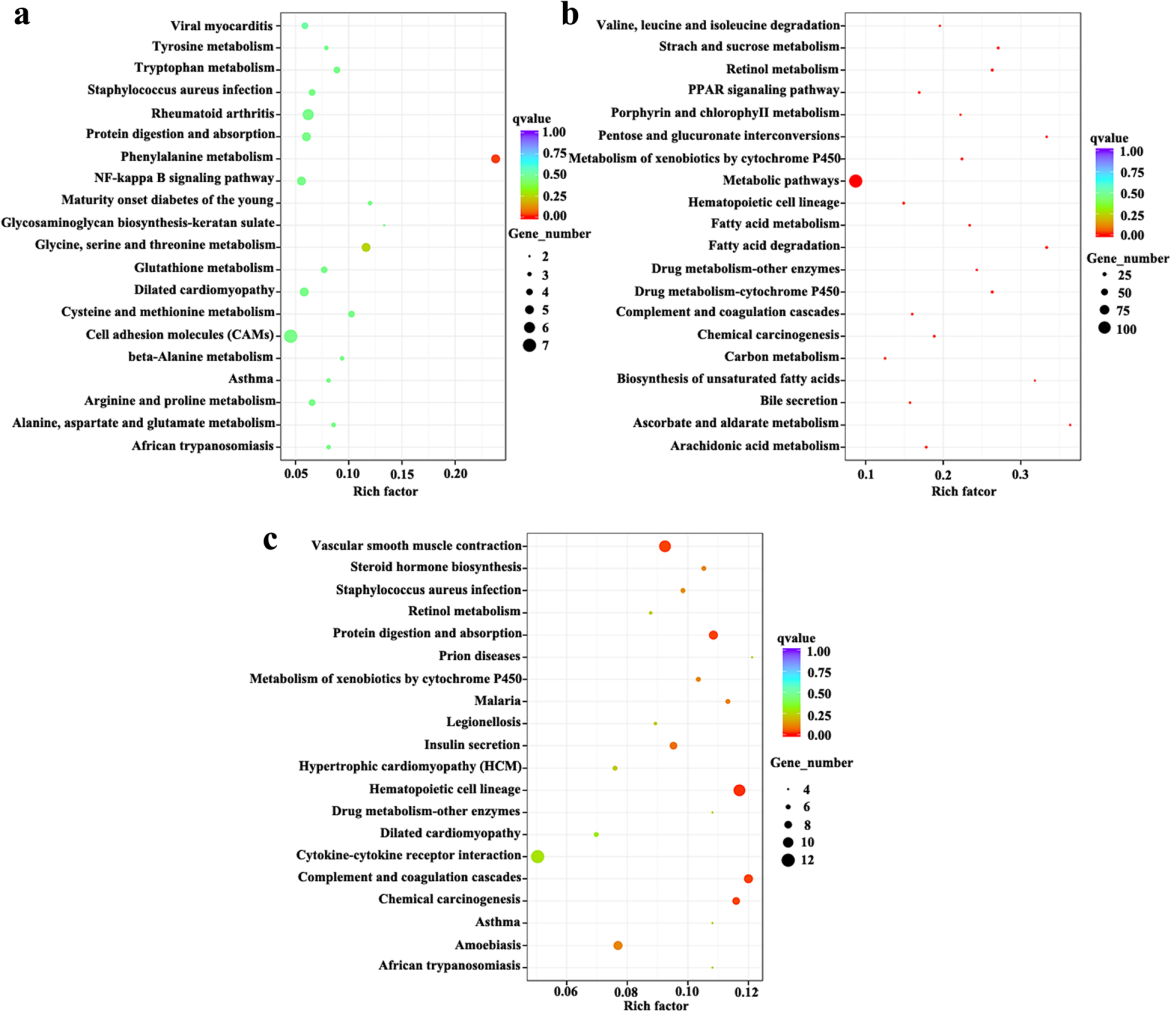
Statistics of KEGG pathway enrichment. The x-axis shows the enrichment factor; the y-axis corresponds to KEGG Pathway. The color of the dot represents q value and size of the dot represents the number of DEGs mapped to the reference pathways. **a**, **b** and **c** represent the top 20 statistics of KEGG pathway enrichment for DEGs observed at 3, 42 and 70 dpi, respectively

KEGG database was used to identify alterations in the biological pathways during *F. gigantica* infection. A total of 501 transcripts were assigned to 183 KEGG pathways at 3 dpi. At 42 dpi 1,194 transcripts were assigned to 229 KEGG pathways, whereas at 70 dpi 639 transcripts were assigned to 193 KEGG pathways. Top 20 most highly represented pathways in each group are shown in Fig. 5.

### Transcription factors

**Fig. 6:**
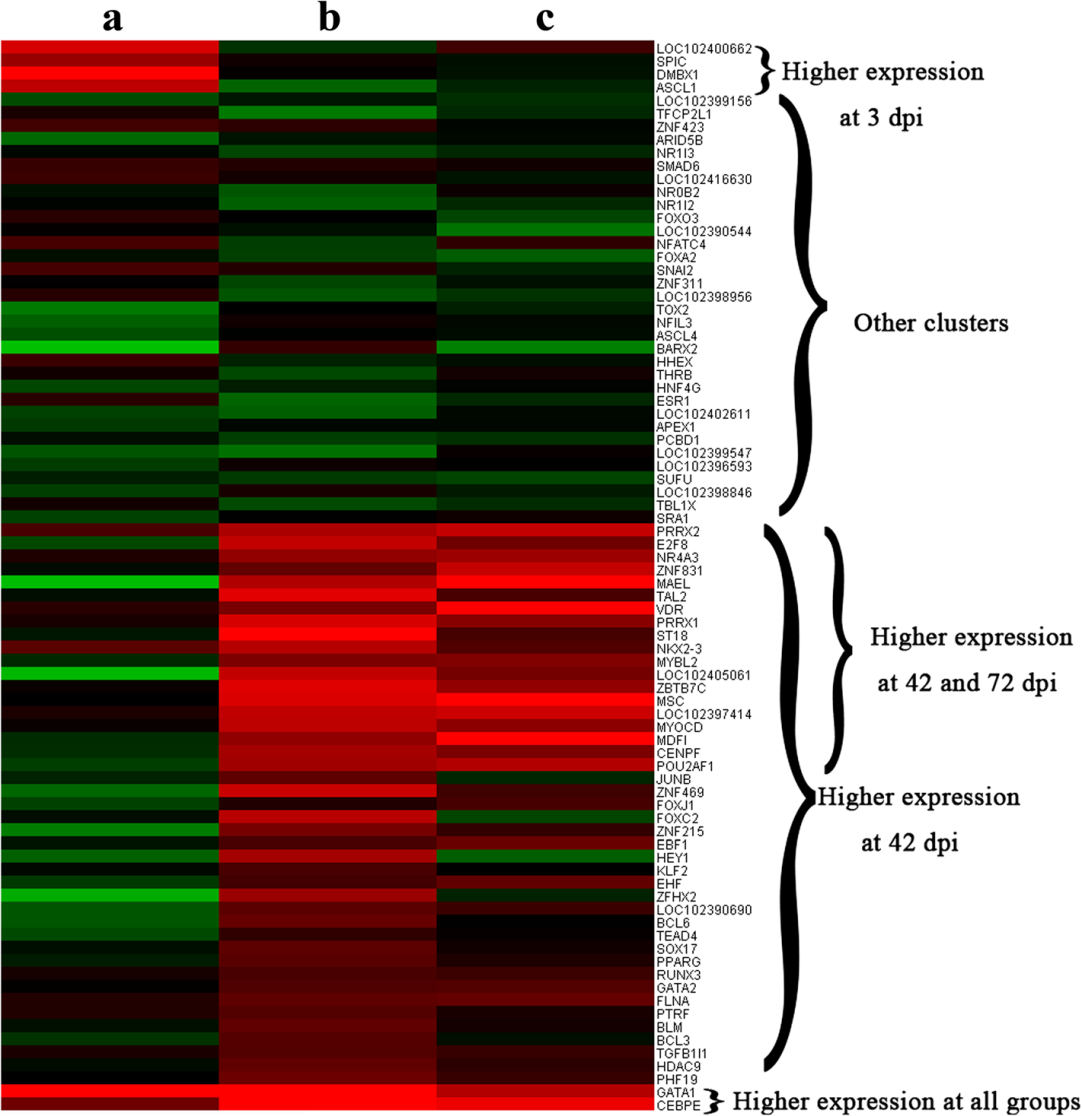
Heatmap of the differentially expressed (DE) transcription factors. **a**, **b** and **c** are differentially expressed transcription factors at 3, 42 and 70 dpi, respectively. The *red* (up) and *green* (down) dots represent the significantly differential expressed transcripts; the *black* represents the transcripts whose expression levels did not reach statistical significance

AnimalTFDB (http://www.bioguo.org/AnimalTFDB/) was used to identify and classify transcription factors in the buffalo genome. As shown in Fig. 6, there are significant differences among the animal groups. Eighty-two differentially expressed transcription factors were identified in infected livers. For example, Smad 6, which inhibits the transforming growth factor beta (TGF-β) signaling pathway, was upregulated at 3 dpi. Also, B cell lymphoma 6 (Bcl6) was downregulated at 3 dpi, but was upregulated at 42 dpi. The differentially expressed transcription factors were classed into 5 clusters according to their expression patterns: (i) four highly expressed genes at 3 dpi; (ii) 19 highly expressed genes at 42 and 72 dpi; (iii) 43 highly expressed genes at 42 dpi; (iv) 2 highly expressed genes in all groups; and (v) 33 genes clustered into other clusters.

## Discussion

In the present study, we employed RNA-Seq Illumina technology to uncover the hepatic transcriptomic changes of buffaloes infected with *F. gigantica*. Specifically, we compared the gene expression patterns of the liver of infected and uninfected buffaloes. GO and KEGG enrichment analyses revealed that experimental infection with *F. gigantica* can influence the expression of genes associated with the host immune response and metabolism. Notably, regulation of some genes indicated potential parasite manipulation to facilitate infection; these included the major histocompatibility complex (MHC) class II (MHC-II) related genes that were repressed, the acute phase protein LBP which was downregulated, modulation of the expression of the transcriptional repressor Bcl6 over the course of infection, upregulation of the transcription factor Smad6, repression of genes involved in the oxidative burst, and finally, modulation of the regenerative response related genes (Brca1 and Blm). The expression patterns of these genes through a time course of 3, 42 and 70 dpi and their relevance to the pathogenesis of *F. gigantica* infection in buffaloes are discussed in the following sections.

### Immune responses

*Fasciola hepatica* infection in lambs can induce a dominant Th2-biased immune response along with suppression of Th1/Th17 responses [21] and can negatively impact Th1 responses to bystander infections, such as during coinfection with *Mycobacterium tuberculosis* [41]. Buffaloes, on the other hand, can exhibit a combination of Th1 and Th2 cytokine expression pattern in response to *F. gigantica* infection [42, 43] or post vaccination with the recombinant fatty acid binding protein (rFABP) and glutathione S-transferase (rGST) protein [44]. Therefore, it is reasonable to expect some differences in the expression patterns of immune response genes that interact with or are stimulated in response to infection with *F. gigantica* and *F. hepatica*. Innate immunity is the first line of defense against *Fasciola* and in the mean time it plays key roles in priming the adaptive immune response [45]. In mammals, antigen processing and presentation are essential for triggering the downstream cellular and/or humoral immune responses [46]. The KEGG results revealed that genes involved in the (MHC-II) pathway were downregulated at 3 dpi, in agreement with others [23], and that genes involved in the MHC-I pathway were upregulated at 42 dpi. Interestingly, at 70 dpi we did not observe any significant alteration in the regulation of MHC-I or MHC-II, suggesting that *F. gigantica* is capable of evading the host immune system. This unique pattern should be further investigated.

The suppression of the MHC-II related genes during early *F. gigantica* infection might correlate with a compromised ability of the MHC class II molecules to present processed *F. gigantica* antigens to CD4(+) T-lymphocytes. Because the stimulation, differentiation, and function of CD4 T cells is central to the development of type I immune responses, any reduction in the interaction between MHC class II molecules and the T cell receptor (TCR) might affect the Th1/Th2 balance, similar to what has been reported in *F. hepatica* infection [21]. A recent study demonstrated that glycoconjugates from *F. hepatica* can induce high levels of IL-10 and IL-4, supporting the role of glycans in the polarization of host immune response toward a Th2/regulatory response *via* induction of IL-10 [25]. Whether the glycosylated molecules of *F. gigantica* could have the same immune-modulatory effect remains to be investigated.

Lipoppolysaccharide binding protein (LBP), acute phase protein, is produced mainly by hepatocytes [47] and plays an important role in lipopolysaccharide (LPS) signaling and innate immunity [48]. LBP can activate Toll-like receptor 4 (TLR4), which mediates the expression of pro-inflammatory cytokines and other immune response related genes [49]. In our study, the downregulation of LBP at 3 dpi, probably mediated by the parasite fatty acid binding protein [49], suggests that host pro-inflammatory responses may be suppressed during early *F. gigantica* infection. This result supports previous findings that showed that *F. hepatica* infection and antigens can suppress Th1 immune responses *in vivo* [41, 50]. Hence, the reduced hepatic production of the LBP and the subsequent suppression of the pro-inflammatory cytokines might be recognized as mechanistically important for survival of the liver flukes in a hostile host environment. Suppression of inflammation has been reported in sheep liver infected with *F. hepatica* [28]. Indeed, the importance of mounting strong Th1 immune responses in the protection of the host against challenge infection has been previously demonstrated in vaccination trials in livestock [51]. While the current literature focussed more on the host immune responses to *Fasciola* has focused on inflammatory responses, our finding suggests that genes that help to keep inflammation in check may also be important in the host response.

Intercellular adhesion molecule-1 (ICAM-1), a member of the immunoglobulin superfamily, is an endothelial- and leukocyte-associated transmembrane protein. ICAM-1 facilitates recruitment of circulating leukocyte (including lymphocytes) to infection/inflamed sites and mediates the interaction between T cells and their target cells [52]. The increase of ICAM-1 expression at 3 dpi is probably beneficial for the adhesion of lymphocytes to the endothelial cells and their migration into the liver, which is a prerequisite of attack of target cells by cytotoxic T lymphocytes. Interestingly, IL-1β was found upregulated at 70 dpi. IL-1β regulates a number of pro-inflammatory genes such as IL-8, a chemokine that attracts neutrophils, and eosinophils, and is involved in antibody-dependent cell-mediated cytotoxicity (ADCC) pathway [53–55], which has a major importance in the defense against *Fasciola* [26]. At 42 dpi, Itgam, which inhibits Ncf1 and Ncf4, leading to impairment of oxidative burst, was significantly upregulated. This suggests that *F. gigantica* alters the expression of genes involved in the oxidative burst process in order to avoid killing by nitric oxide (NO), which is considered as a defense mechanism against infection [56]. This finding is consistent with the previous result that NO production and nitric oxide synthase 2 (NOS2) expression are downregulated when monocyte-derived macrophages of human origin were exposed to *F. hepatica* fatty acid binding protein, known as Fh12 [57]. Our observation is also in agreement with previous studies that reported inability of ovine macrophages to generate NO when incubated with newly excysted juveniles of *F. hepatica in vitro* [58] and the significant downregulation of NOS2 gene, encoding inducible nitric oxide synthase (iNOS), which converts arginine into citrulline and NO during the acute and chronic stages of ovine *F. hepatica* infection [21].

We also identified DEGs involved in processes associated with the regulation of Th2 cell differentiation and B-cell activation. We also found, at 3 dpi, upregulation of immunoglobulin variable gene (Ighv1s28, Ig heavy chain Mem5), which inhibits Pro-B cell differentiation to Pre-B1 cell. At 42 dpi, Cd3e, Zap70 and Il-17r genes, which are involved in inhibiting the hematopoietic stem cell (HSC) differentiation, were upregulated. Our study also identified DE transcription factors, such as the transcriptional repressor Bcl6, which is essential for the formation of T-follicular helper (Tfh) cells [59–61], which facilitates T cell-dependent B cell differentiation and antibody responses [62]. Bcl6 was downregulated at 3 dpi, but was upregulated in 42 dpi, suggesting that *F. gigantica* infection can modulate Bcl6 expression (a traditional regulator of a Th2 response) over the course of infection. This finding is also in agreement with previous work [28] and is consistent with the literature where changing from Th1 to Th2 response occurs as infection establishes, correlating with the development of adaptive B cell response and the generation of *Fasciola*-specific antibodies within 4 weeks of infection [45]. The transcription factor Smad6 is an inhibitor of TGF-β signaling pathway [63], which plays a key role in fibrosis during *F. hepatica* infection [64] and can suppress the growth and self-renewal of hepatic progenitor cells [65]. In our study, Smad6 was found upregulated at 3 dpi, suggesting that Smad6 may play a role in controlling fibrosis during the early stage of *F. gigantica* infection. A previous study reported a similar finding in PBMC isolated from sheep infected with *F. hepatica* where the expression level of the inhibitory-Smad protein, Smad7, was upregulated, which has been hypothesized to play a role in limiting the fibrosis formation during acute and chronic stage of infection [21].

In contrast, increased levels of PBMC-derived TGF-β1 have been observed in early phases of the infection with *F. hepatica* in cattle [22]. These findings indicate that molecules of the TGF-β-pathway can potentially exacerbate and ameliorate the liver fibrotic process depending on the stage of the infection, host species and *Fasciola* species causing the infection.

### Metabolic dysregulation

Liver is a very important metabolic and drug clearance organ because changes in the activities or regulation of hepatic drug metabolizing enzymes can alter clearance of chemical compounds. Previous reports indicated that *F. hepatica* infection can induce alterations in the mitochondrial electron transport chain and the enzymes that are responsible for drug metabolism in the liver [28, 66, 67]. Several hepatic enzymes known to play key roles in the mammalian metabolic and clearance processes, including Flavin monooxygenase (FMO), carboxylesterases (CES), members of cytochrome P450 enzyme, aldehyde dehydrogenase (ALDH), glutathione *S*- transferase (GST), and paraoxonase (PON), were found to be affected by *F. gigantica* infection in our study. For example, the expression level of Pon1 in infected liver was 2.2-fold higher compared to the control liver at 3 dpi. At 42 and 70 dpi, infected buffaloes had lower levels of Pon3 (58%) and Ces2 (49%), respectively. At 3 dpi, the mRNA level of Aldh1a1 was 1.6-fold higher than the corresponding control. Other aldehyde dehydrogenases in the infected liver had lower levels [e.g. Aldh3a2 (43%), Aldh1l1 (51%), Aldh4a1 (57%) at 42 dpi and Aldh1l1 (50%) at 70 dpi]. Also, Fmo3 and Fmo5 showed decreased levels (60%) in infected liver samples. The mRNA levels of GST decreased in infected livers at 42 dpi [e.g. Gsta2 (50%) and MGst1 (58%)] and at 70 dpi [e.g. Gsta1 (68%)].

Cytochrome P450 is a very important drug metabolizing enzyme. KEGG analysis revealed that in the cytochrome P450 pathway there are 2 upregulated transcripts (Gstm1 and Gsta3) at 3 dpi, 13 downregulated transcripts (Fmo3, Fmo5, UDP-glucuronosyltransferase 2b4, UDP glucuronosyltransferase 2 family, UDP-glucuronosyltransferase 2C1, Ugt1a6, Ugt2b17, Ugt1a1, Adh5, Gsta2, Gsto1, Mgst1, Maob) at 42 dpi and 4 downregulated transcripts (Ugt2a1, Mgst1, Gsta5, Gsta3) at 70 dpi. The downregulation of cytochrome P450 genes is consistent with previous results [68]. The alterations of these enzymes suggest that infection of buffaloes with *F. gigantica* infection can modulate drug pharmacokinetics, and this can vary over the course of infection.

### Genomic responses

The breast cancer 1 early onset (Brca1), which is a tumor suppressor involved in cellular functions related to cell replication and DNA synthesis was found downregulated at 3 and 42 dpi. Blm, coding blooms syndrome helicase which is a member of the RecQ family of superfamily 2 helicases and plays critical role in the maintenance of genome stability, was found upregulated at 42 dpi. The clinical relevance of the downregulation of Brca1 or upregulation of Blm is still unknown, but it is likely that Bcra1-mediated cell proliferation and Blm-mediated genome integrity play a role in the regenerative response of the liver to heal the damaged tissue caused by *F. gigantica* infection. A similar observation was reported in sheep livers infected with *F. hepatica* where genes associated with host cell cycle and mitosis were found significantly upregulated [28].

### Bile secretion

Because they live and induce pathological lesions in the bile duct, *F. gigantica* flukes are expected to interfere with bile synthesis and secretion. Indeed, alteration in the bile production has been reported in *F. hepatica* infected sheep [69]. The downregulation of the Na^+^ and D-glucose transfer (Sglt1) gene, expressed at the cholangiocyte apical plasma membrane, at 3 dpi can decrease the apical uptake of glucose from the bile and this could affect the biliary osmolarity. Genes related to the synthesis of carbonic acid (H_2_CO_3_) from CO_2_ and H_2_O, which facilitates the excretion of bile acid and glutathione (GSH) through kidney were upregulated, potentially causing a reduction in the amount of bile in the biliary duct.

## Conclusions

Using RNA sequencing technology, we characterized transcriptome profiles of water buffalo liver during experimental *F. gigantica* infection. Comparing the infection groups to the mock groups, 496, 880 and 441 significantly DEGs were identified at 3, 42 and 70 dpi, respectively. Infected liver showed alterations in the expression of genes involved in immune responses, hepatic drug metabolism, regenerative response, and bile secretion. Several pathways, such as the MHC antigen processing and presentation, TLR4 signalling, TGF-β signalling, and cytochrome P450 pathway, have been altered by *F. gigantica* infection. These findings suggest that *F. gigantica* can modulate host immunity and inflammatory pathways in order to facilitate its survival within the host. A better understanding of the immunopathological mechanisms of *F. gigantica* infection may provide new preventive and therapeutic strategies for the control of fasciolosis in buffaloes.

## Additional files

Additional file 1:
**Figure S1.** Liver of an infected buffalo showing adult *Fasciola gigantica* fluke *in situ*. (TIF 1827 kb)
Additional file 2:
**Figure S2.** Adult *Fasciola gigantica* fluke isolated from the liver of an infected buffalo. (TIF 6665 kb)
Additional file 3:
**Table S1.** Summary of all data obtained from all samples in the present study. (DOC 46 kb)

## Abbreviations

ADCC: antibody-dependent cell-mediated cytotoxicity
ALDH: aldehyde dehydrogenase
AR: anthelmintic resistance
CES: carboxylesterases
DEGs: differentially expressed genes
dpi: days post-infection
FMO: Flavin monooxygenase
FPKM: Fragments per kilobase of transcript sequence per millions base pairs sequenced
GST: glutathione *S*- transferase
HSC: hematopoietic stem cell
ICAM: intercellular adhesion molecule
LBP: lipopolysaccharide binding protein
MHC: major histocompatibility complex
PBMCs: peripheral blood mononuclear cells
PBS: phosphate buffered saline
PON: paraoxonase
RNA-seq: RNA sequencing
TGF-β: transforming growth factor beta
TLR4: toll-like receptor 4
