# Supplementary figures and images for: Transcriptomic responses of water buffalo liver to infection with the digenetic fluke Fasciola gigantica
Source: Parasit Vectors. 2017 Feb 1;10:56. doi: 10.1186/s13071-017-1990-2 (PMC5286860; doi:10.1186/s13071-017-1990-2)

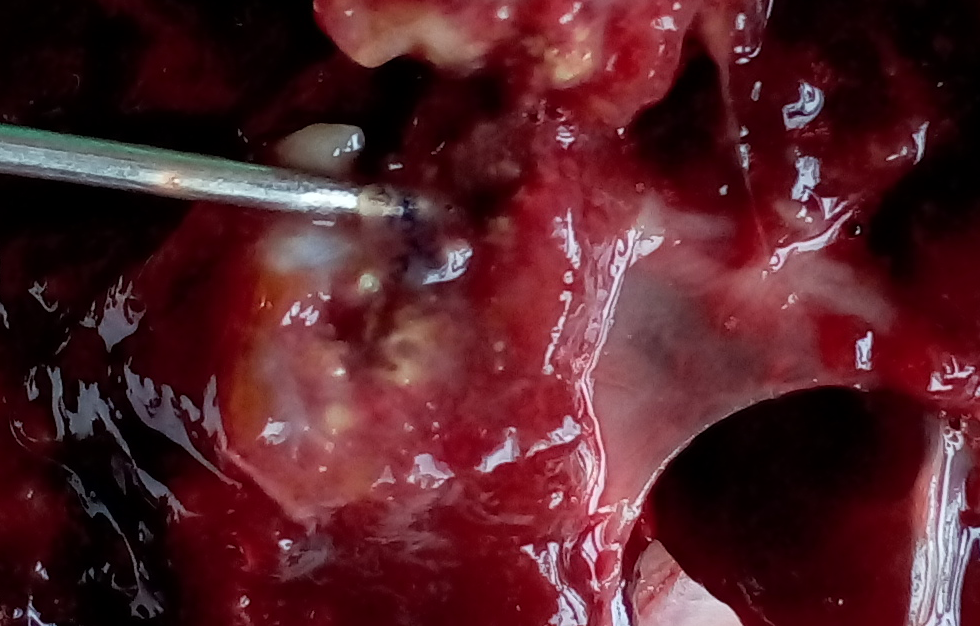

Supplement: Additional file 1: — Figure S1. Liver of an infected buffalo showing adult Fasciola gigantica fluke in situ. (TIF 1827 kb) [file 13071_2017_1990_MOESM1_ESM.tif]

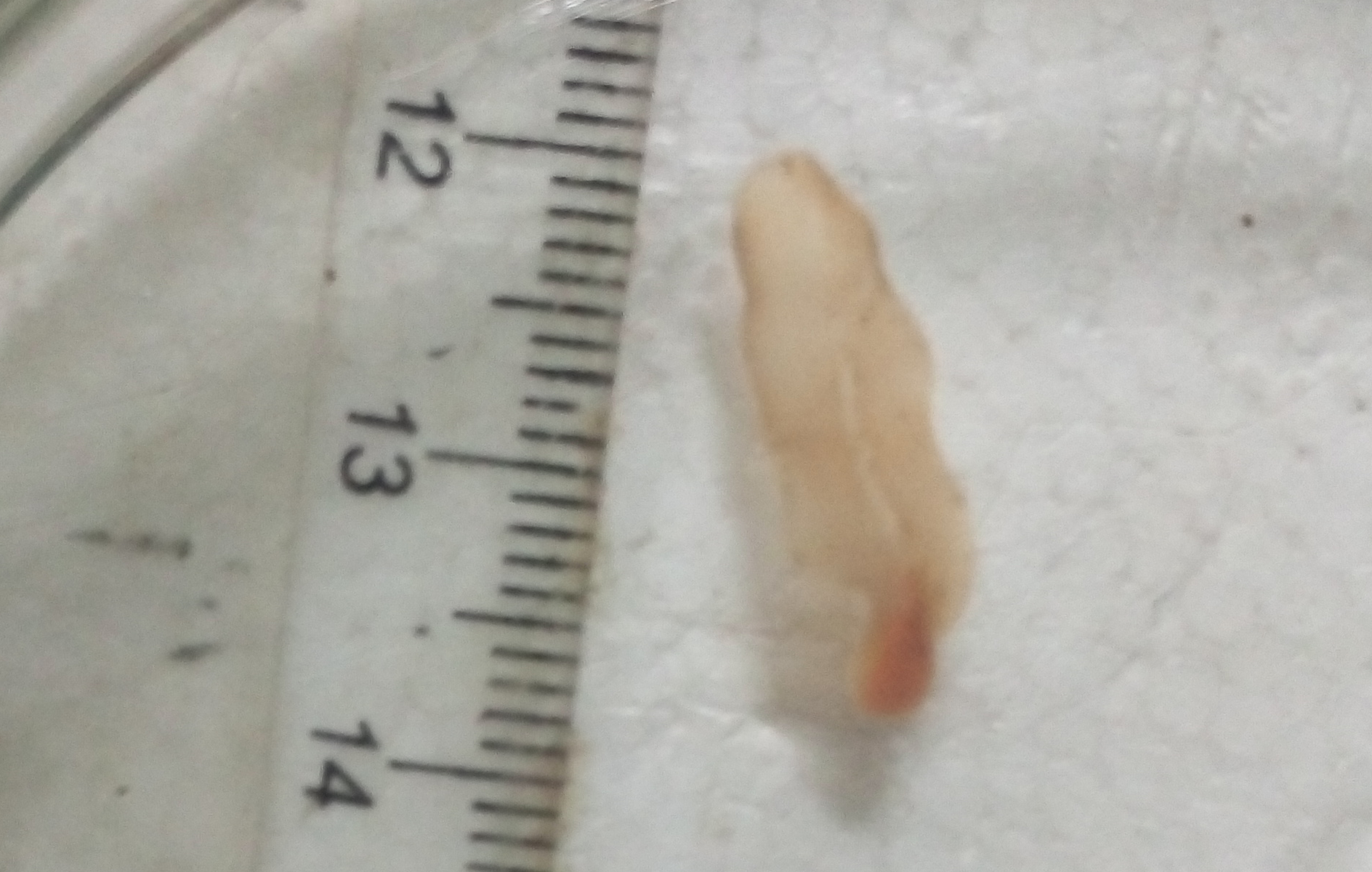

Supplement: Additional file 2: — Figure S2. Adult Fasciola gigantica fluke isolated from the liver of an infected buffalo. (TIF 6665 kb) [file 13071_2017_1990_MOESM2_ESM.tif]
