# Supplementary material for: Transcriptomic responses of water buffalo liver to infection with the digenetic fluke Fasciola gigantica
Source: Parasit Vectors. 2017 Feb 1;10:56. doi: 10.1186/s13071-017-1990-2 (PMC5286860; doi:10.1186/s13071-017-1990-2)
Supplement: Additional file 3: — Table S1. Summary of all data obtained from all samples in the present study. (DOC 46 kb) [file 13071_2017_1990_MOESM3_ESM.doc]

**Table S1** Summary of all data obtained from all samples in the present study

| **Animal groups ^a^** | **Sample**  **ID** | **Raw reads** | **Clean reads** | **Clean bases** | **Error rate (%)** | **Q20^b^ (%)** | **Q30^c^ (%)** | **GC content (%)** |
| --- | --- | --- | --- | --- | --- | --- | --- | --- |
| Control  3dpi | A2C1 | 58779918 | 57771238 | 8.67G | 0.01 | 98.11 | 95.29 | 49.42 |
|  | A2C2 | 61679484 | 60620900 | 9.09G | 0.01 | 98.15 | 95.39 | 49.04 |
|  | A2C3 | 61673824 | 60563744 | 9.08G | 0.01 | 98.03 | 95.10 | 49.58 |
| Infected  3dpi | A2T1 | 69326390 | 68070124 | 10.21G | 0.01 | 98.07 | 95.23 | 49.86 |
|  | A2T2 | 54749108 | 53774222 | 8.07G | 0.01 | 98.03 | 95.13 | 48.93 |
|  | A2T3 | 68789606 | 67574524 | 10.14G | 0.01 | 97.87 | 94.78 | 49.65 |
| Control  42 dpi | A5C1 | 72092886 | 70817946 | 10.62G | 0.01 | 98.07 | 95.22 | 48.98 |
|  | A5C2 | 55104034 | 54162440 | 8.12G | 0.01 | 98.08 | 95.20 | 49.85 |
|  | A5C41 | 70822610 | 69535842 | 10.43G | 0.01 | 98.14 | 95.39 | 49.15 |
| Infected  42 dpi | A5T30 | 59280312 | 56628564 | 8.49G | 0.01 | 97.66 | 94.49 | 50.62 |
|  | A5T32 | 62970852 | 60404926 | 9.06G | 0.01 | 97.49 | 94.04 | 48.99 |
|  | A5T33 | 59535514 | 57077258 | 8.56G | 0.01 | 97.59 | 94.29 | 50.42 |
| Control  70 dpi | A6C1 | 67748128 | 66351444 | 9.95G | 0.01 | 97.97 | 95.04 | 50.18 |
|  | A6C2 | 60159308 | 58950944 | 8.84G | 0.01 | 97.87 | 94.77 | 50.04 |
|  | A6C3 | 59653148 | 58543470 | 8.78G | 0.01 | 97.91 | 94.85 | 49.45 |
| Infected  70 dpi | A6T3 | 67287870 | 66019464 | 9.9G | 0.01 | 97.75 | 94.55 | 48.94 |
|  | A6T4 | 70435824 | 69033572 | 10.36G | 0.01 | 97.88 | 94.83 | 49.19 |
|  | A6T5 | 72236836 | 70989232 | 10.65G | 0.01 | 97.97 | 95.02 | 50.07 |

^a^ At each of the indicated time points, 3, 42 and 70 day after infection, there are 3 control and 3 infected buffaloes

^b^ The percentage of nucleotide bases with a Phred base quality score (Q score) value > 20, i.e., the proportion of read bases whose error rate is less than 1%

^c^ The percentage of nucleotide bases with a Phred base quality score (Q score) value >30, i.e., the proportion of read bases whose error rate is less than 0.1%
